# Alterations in Blood Markers, Rumen Fermentation, and Microbiota Due to Heat Stress in Holstein Cows During the Dry Period and Early Lactation

**DOI:** 10.3390/ani16111682

**Published:** 2026-05-30

**Authors:** Ye Pyae Naing, Seon-Ho Kim

**Affiliations:** Ruminant Nutrition and Anaerobe Laboratory, Department of Animal Science and Technology, Sunchon National University, Suncheon 57922, Republic of Korea; yepyaenaing.scnu@gmail.com

**Keywords:** heat stress (HS), blood markers, rumen, microbiota, dairy cows

## Abstract

In this study, physiological and microbial responses under heat stress conditions were evaluated by analyzing blood biochemical parameters, rumen fermentation characteristics, and rumen microbiome (bacterial community composition) in Holstein dairy cows during the dry period and early lactation under summer conditions in Korea. Fourteen cows were observed during the hot summer month (from the first to the third week of August), with the temperature-humidity index (THI) recorded in the barns during the dry and early lactation periods being 80.80 and 81.66, respectively. Blood and rumen fluid samples were collected to evaluate physiological responses and changes in blood parameters, rumen fermentation, and rumen microbial composition. Blood analysis revealed significant variations between the two stages. Early lactating cows exhibited lower glucose, blood urea nitrogen, and cholesterol levels but higher ketone and aspartate aminotransferase levels, indicating increased energy demands and protein metabolism. A complete blood count showed reduced red blood cell count, hematocrit, and hemoglobin levels during the early lactation period, whereas white blood cell counts increased. The levels of heat shock proteins (HSPs), such as HSP27, HSP70, and HSP90, also differed significantly. Rumen fermentation analysis revealed lower ammonia nitrogen concentrations but significantly higher propionate and total volatile fatty acid concentrations during the early lactation period, indicating adaptive changes in rumen function. Rumen microbial analysis revealed significant differences in bacterial diversity and composition. Early lactation cows exhibited relatively high abundances of Bacteroidota and *Prevotella*, whereas the dry period was dominated by Clostridia and Eubacteriales. Network analysis highlighted shifts in microbial interactions, with specific keystone species identified at each stage. These findings suggest distinct physiological and rumen microbial adaptations in response to HS, with early lactation characterized by heightened metabolic demands and significant shifts in rumen bacterial communities. Such insights could inform tailored management strategies to mitigate the impact of HS on dairy cows during their critical production stages.

## Simple Summary

Heat stress during hot summer conditions can affect the health and productivity of dairy cows, especially during important stages such as the dry period and early lactation. This study aimed to understand how high temperatures influence body responses, blood health, digestion, and gut microorganisms in Holstein dairy cows in Korea. Fourteen cows were observed during August, when heat levels were high. Blood and rumen (forestomach) samples were collected to evaluate changes in metabolism and digestion. The results showed that cows in early lactation had lower energy-related substances in their blood but higher markers indicating increased energy use and stress. Changes in blood cell counts also suggested differences in immune response. In the rumen, cows in early lactation showed improved production of certain fermentation products that provide energy, along with shifts in the types of microorganisms present. These findings indicate that cows adapt differently to heat stress depending on their stage of production, with early lactation cows facing greater metabolic challenges. Understanding these changes can help farmers develop better feeding and management strategies to reduce the negative effects of heat stress and improve animal health and productivity.

## 1. Introduction

The transition from the dry period to early lactation is a critical stage for Holstein dairy cows, during which heat stress (HS) poses significant challenges. It alters metabolic processes, leading to changes in biomarker levels that reflect the health and productivity of the cows [1]. Elucidating the variations in the levels of these biomarkers is essential for developing effective management strategies to improve cow performance and health under HS [2]. HS, indicated by a temperature-humidity index (THI) exceeding 72, detrimentally affects the health and productivity of dairy cows. High THI levels increase the physiological heat load on cows, challenging their ability to regulate body temperature [3]. As ambient temperatures increase, heat-stressed cows experience elevated body temperatures along with altered metabolic and reproductive functions. High THI levels also adversely affect fertility in dairy cows, causing delayed first estrus postpartum and relatively long calving intervals. Cows exposed to a THI exceeding 72 for seven days before calving experience a concerning decline in their reproductive performance, characterized by increased services per conception [4].

Rumen fermentation is crucial for dairy cows, enabling them to digest fibrous plant materials into volatile fatty acids (VFAs), which serve as their primary energy source [5]. The rumen microbiome plays a critical role in the health and productivity of dairy cows by affecting their digestive efficiency and nutrient absorption [6]. During the transition phase from the dry period to early lactation, significant physiological changes occur that require optimal rumen fermentation and microbial stability. The transition period is characterized by a negative energy balance and an increased risk of metabolic disorders, where the energy requirements of a cow suddenly increase because of milk production, making an effective management of the rumen microbiome critical during this period [7,8]. HS presents formidable challenges for rumen function and microbial composition. It can alter microbial community dynamics, reduce feed intake, and affect fermentation processes, leading to diminished energy production and poor overall health of dairy cows [9]. High temperatures during HS conditions negatively affect the rumen microbiome, shifting populations toward an increased abundance of lactic acid-producing bacteria while reducing fiber-digesting bacteria, which disrupts normal fermentation processes [10]. Overall, the interplay between rumen fermentation dynamics, bacterial community composition, and environmental stressors, such as heat, is essential to ensure the health and productivity of dairy cows, particularly during the transition to lactation [9,11].

The transition from the dry period to early lactation is a pivotal stage in dairy cows, marked by intense physiological and metabolic adjustments to support milk production. HS exacerbates these demands, impacting metabolic function, immune response, and microbial balance, which can adversely affect cow health and productivity. Although HS has been shown to alter blood biochemical parameters, rumen fermentation, and microbial communities, its differential impact across the dry and early lactation periods of cows remains underexplored. Therefore, this study aimed to examine the physiological and microbial adaptations to HS in Holstein cows during these critical stages. Specifically, we investigated changes in blood biochemical profiles, heat shock protein (HSP) concentrations, rumen fermentation parameters, and ruminal microbiota composition to provide insights into how HS impacts the metabolic and microbial dynamics in dairy cows. The findings of this study may contribute to refining dairy management practices to alleviate HS effects and promote the health and productivity of dairy cows during periods of elevated environmental stress.

## 2. Materials and Methods

### 2.1. Animal Care

All experimental procedures were conducted in accordance with the Animal Experimental Guidelines provided by the Sunchon National University Institutional Animal Care and Use Committee of the Republic of Korea. The experimental protocol was approved by SCNU-IACUC (approval number: SCNU IACUC-2025-15).

### 2.2. Animals, Diets, and Experimental Design

The experiments were performed on Holstein dairy cows during both the dry period and early lactation stage. Fourteen Holstein dairy cows were used in this study: seven cows in the dry period (an average of 21 ± 5 days before the expected calving date) and seven cows in the early-lactation period (first 30 ± 10 days in milk (DIM)). The cows were grouped according to physiological stage rather than experimental treatment. The study was conducted during the hot summer month (from the first week to the third week of August) under naturally occurring heat stress conditions. Although both groups were evaluated during the same summer period, slight differences in temperature humidity index (THI) values were observed between the dry-period and early-lactation groups due to variations in environmental conditions during the respective sampling times. The THI recorded in the barns during the dry period was 80.80 (temperature: 28.25 °C; humidity: 85.59%), whereas during the early-lactation period it was 81.66 (temperature: 29.53 °C; humidity: 76.58%). According to the THI classification for Holstein dairy cows, THI values above 80 were categorized as severe heat stress conditions. In the third week of both the dry and early-lactation stages, the criteria used to determine environmental heat stress conditions for the dry-period and early-lactation groups were based on THI [12], calculated using the following formula:

THI = (0.8 × ambient temperature) + [(% relative humidity/100) × (ambient temperature − 14.4)] + 46.4

All dairy cows were fed twice daily (at 06:00 and 17:00) with a total mixed ration (TMR) diet. Rumen fluid and blood samples were collected from the Holstein dairy cows.

### 2.3. Sample Collection

**Figure 1:**
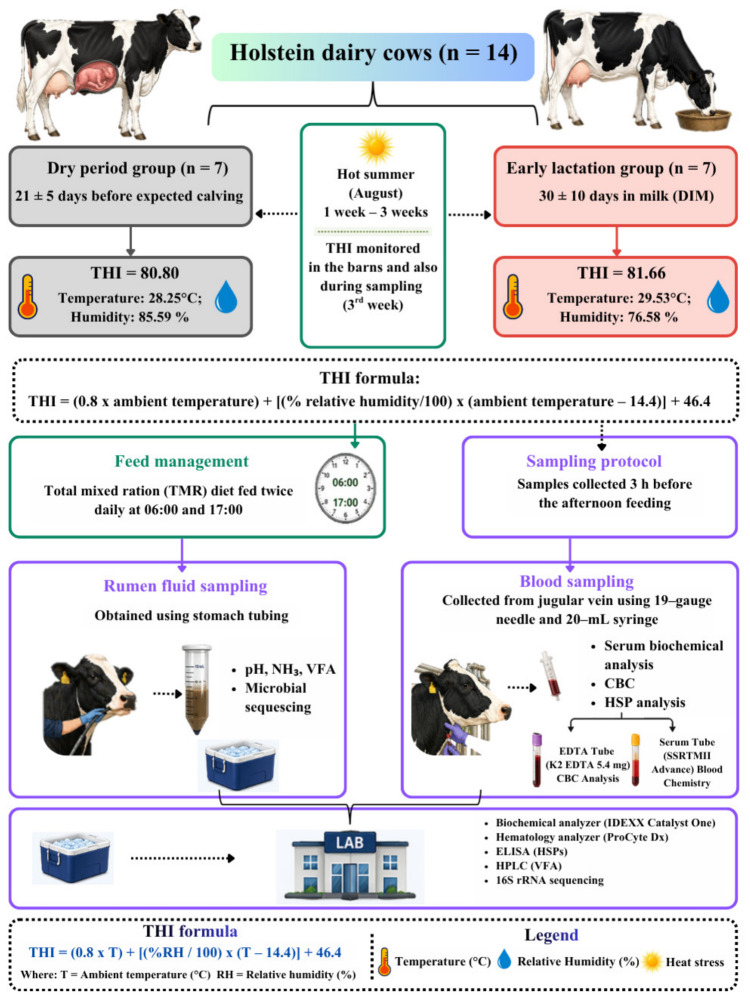
Experimental design, environmental conditions (THI), and sampling procedures in Holstein dairy cows during the dry period and early lactation under heat stress conditions.

Samples were collected 3 h prior to afternoon feeding to evaluate the composition of the baseline diet. All dairy cows were fed twice daily (at 06:00 and 17:00) with a total mixed ration (TMR) diet. Rumen fluid and blood samples were collected from the Holstein dairy cows Figure 1.

The ingredient profile and chemical composition (%) of the total mixed ration (TMR) are presented in Table 1. Rumen fluid was collected from all 14 Holstein dairy cows using a stomach tube and transferred into individual 50 mL conical centrifuge tubes, followed by storage in an ice chest. Blood samples were drawn from the jugular vein using a 19-gauge needle attached to a 20 mL syringe for hematological analysis. Approximately 5 mL of each blood sample was placed into a Vacutainer tube for subsequent determination of blood biochemical parameters and complete blood count (CBC). The BD Vacutainer^®^ K2 EDTA 5.4 mg (Becton Drive, Franklin Lakes, NJ, USA) and BD Vacutainer^®^ SSRTM II Advance (Belliver Industrial Estate, Belliver Way, Robrough, Plymouth, UK) containers were used for this purpose. The blood samples were immediately placed in an ice box and transported to the laboratory for further processing.

### 2.4. Blood Biochemistry Analysis

To obtain serum, blood samples were centrifuged at 4 °C for 10 min at 1800× *g*. The resulting supernatant was carefully separated and stored in clean tubes at −20 °C for subsequent analysis. Serum biochemical parameters were determined using a Catalyst One™ Chemistry Analyzer (IDEXX Laboratories, Inc., Westbrook, ME, USA), including aspartate aminotransferase (AST), blood urea nitrogen (BUN), calcium, cholesterol, magnesium, phosphorus, total bilirubin, and total protein. In addition, glucose and β-hydroxybutyrate (BHBA) concentrations were measured using a FreeStyle Optium Neo H system (Abbott, Precision Xtra^®^, Alameda, CA, USA). A portion of the serum samples was also submitted to the Department of Pharmacy, Sunchon National University, Republic of Korea, for metabolomics analysis.

### 2.5. Complete Blood Count (CBC) Analysis

The complete blood count (CBC) of samples transported to the laboratory was analyzed using an IDEXX ProCyte Dx™ hematology analyzer (Westbrook, ME, USA). The instrument measured multiple hematological indices, including red blood cell (RBC) and white blood cell (WBC) counts, hemoglobin concentration, hematocrit (HCT), mean corpuscular volume (MCV), mean corpuscular hemoglobin (MCH), and mean corpuscular hemoglobin concentration (MCHC). Additional parameters included red cell distribution width (RDW), reticulocyte count (RETIC), platelet count, mean platelet volume, platelet distribution width, and immature granulocyte count.

### 2.6. Heat Shock Protein (HSP) Analysis

Serum concentrations of HSP27, HSP70, and HSP90 were determined using commercially available bovine-specific enzyme-linked immunosorbent assay (ELISA) kits purchased from MyBiosource, Inc. (San Diego, CA, USA). All analyses were performed according to the manufacturer’s protocol.

### 2.7. Analysis of Rumen Fermentation Parameters

The rumen fluid samples were analyzed using advanced equipment for pH, VFA, and ammonia nitrogen (NH_3_-N). A SevenCompact^TM^ pH/Ion meter (Mettler-Toledo, Greifensee, Switzerland) was used to measure the pH of the samples immediately after collection, following which the fluid samples were stored at −80 °C in cryotubes. The frozen samples were thawed and centrifuged, and the supernatants were used for evaluating NH_3_-N and VFA concentrations. The concentration of NH_3_-N was determined at 630 nm using a Libra S22 spectrophotometer (Biochrom Ltd., Cambridge, UK), whereas high-performance liquid chromatography was used for VFA concentration analysis, along with an ultraviolet detector, a specific column, and a solvent [13].

### 2.8. DNA Extraction and Metataxonomic Analysis

DNA was extracted from rumen fluid samples using the PowerSoil^®^ DNA Isolation Kit (MoBio Laboratories, Carlsbad, CA, USA) according to the manufacturer’s instructions. The extracted samples were submitted to Macrogen Inc. (Seoul, Republic of Korea) for 16S rRNA amplicon sequencing. DNA concentration was quantified using PicoGreen and NanoDrop assays. Amplicon libraries were constructed following the Illumina 16S Metagenomic Sequencing Library Preparation protocol. The V3–V4 region of the bacterial 16S rRNA gene was amplified using the universal primers 341F (5′-CCTACGGGNGGCWGCAG-3′) and 805R (5′-GACTACHVGGGTATCTAATCC-3′). Amplicon libraries were prepared according to the Illumina MiSeq protocol. After demultiplexing and adapter trimming using Cutadapt (v3.2), amplicon sequence variants (ASVs) were generated with DADA2 (v1.18.0) [14], incorporating error correction and read truncation. Microbial community analysis was conducted using QIIME (v1.9) [15], and each ASV was aligned to the closest reference sequence in the NCBI 16S Microbial Database using BLAST+ (v2.9.0) [16]. The metadata, ASV, and taxonomy tables were then uploaded to MicrobiomeAnalyst [17,18,19], where low-abundance and low-variance features were filtered using default settings. The filtered dataset was subsequently normalized using total sum scaling.

### 2.9. Statistical Analysis

Statistical Analysis Systems (SAS) software version 9.4 (SAS Institute Inc., Cary, NC, USA) was used to analyze rumen fermentation and blood composition parameters between the two experimental stages. Data were statistically evaluated using the PROC GLIMMIX procedure for a completely randomized design. Differences between the two groups were assessed using analysis of variance (ANOVA) followed by pairwise comparisons. Statistical significance was declared at *p* < 0.05. For microbiome analysis, alpha diversity indices, including observed ASVs, Chao1, Shannon, and Simpson indices, were calculated to evaluate microbial diversity. These indices were compared using GraphPad Prism version 8.0.2. Beta diversity was assessed to examine differences in community structure using ANOVA, permutational multivariate analysis of variance (PERMANOVA), and permutational analysis of multivariate dispersion (PERMDISP), based on Bray–Curtis distance matrices. These approaches were used to evaluate variation in microbial community composition and dispersion between the dry and early-lactation periods. Microbial composition was visualized using stacked bar plots at both phylum and species levels to show relative abundance patterns. Linear discriminant analysis effect size (LEfSe) was applied to identify potential biomarkers associated with each physiological stage. In addition, microbial interaction patterns and co-occurrence networks were explored to assess changes in ruminal species interactions between the dry and early-lactation stages.

## 3. Results

### 3.1. Comparison of Blood Biochemical Parameters Between Dry and Early Lactation Under Heat Stress Conditions

**Figure 2:**
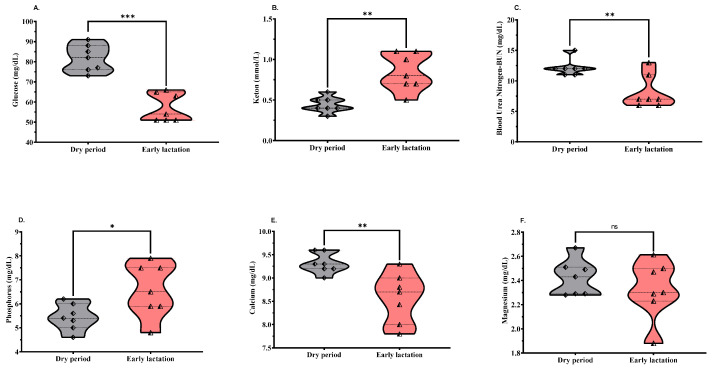

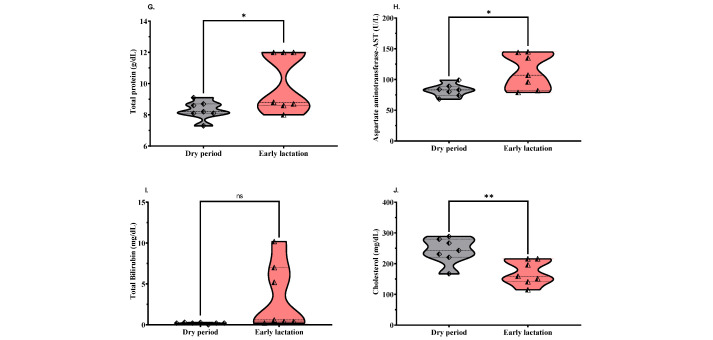
Differences in blood biochemical parameters, including (**A**) glucose, (**B**) ketone, (**C**) blood urea nitrogen—BUN, (**D**) phosphorus, (**E**) calcium, (**F**) magnesium, (**G**) total protein, (**H**) aspartate aminotransferase—AST, (**I**) total bilirubin, and (**J**) cholesterol levels, during heat stress (HS) conditions between the dry and early lactation periods of Holstein cows. Statistical significance is considered at a *p*-value less than 0.05 (*p* < 0.05). *** *p* < 0.0001; ** *p* < 0.001; * *p* < 0.01; ns, *p* > 0.0.

Blood biochemical parameters were analyzed using PROC GLIMMIX, and differences between dry and early-lactation stages were considered significant at *p* < 0.05. Glucose levels were significantly lower during the early lactation period (57.29 ± 2.66 mg/dL) compared with those in the dry period (81.71 ± 2.52 mg/dL), *p* < 0.0001 (Figure 2A), whereas ketone levels were higher in the early lactation stage (0.84 ± 0.09 mmol/L) than those in the dry period (0.44 ± 0.04 mmol/L), *p* = 0.0012 (Figure 2B). In addition, BUN levels were lower during the early lactation period (8.14 ± 1.03 mg/dL) than those in the dry period (12.14 ± 0.51 mg/dL), *p* = 0.0046 (Figure 2C). Similarly, Ca levels were higher in the dry period (9.31 ± 0.08 mg/dL) compared to early lactation (8.85 ± 0.20 mg/dL), *p* = 0.0055 (Figure 2E), whereas total protein was higher in the early lactation period (10.01 ± 0.71 g/dL) than that in the dry period (8.30 ± 0.22 g/dL), *p* = 0.0393 (Figure 2G). AST levels were elevated in the early lactation period (112.57 ± 10.81 U/L) compared with those in the dry period (82.43 ± 3.80 U/L), *p* = 0.0220 (Figure 2H). In addition, bilirubin levels were higher during the early lactation period (3.43 ± 1.53 mg/dL) than those in the dry period (0.23 ± 0.02 mg/dL), but the difference was not significant between the two stages, *p* = 0.0813 (Figure 2I). Cholesterol levels showed an opposite trend, being significantly lower in the early lactation period (170.57 ± 14.84 mg/dL) than in the dry period (242.57 ± 15.78 mg/dL) (Figure 2J), *p* = 0.0061. Moreover, phosphorus levels were higher in the early lactation period (6.57 ± 0.42 mg/dL) than those in the dry period (5.44 ± 0.21 mg/dL), *p* = 0.0342 (Figure 2D). Mg levels did not differ significantly between the two stages, with values of 2.33 ± 0.09 mg/dL during early lactation and 2.42 ± 0.06 mg/dL during the dry period, *p* = 0.3798 (Figure 2F).

### 3.2. Comparison of Complete Blood Count (CBC) Parameters Between Dry and Early Lactation Under Heat Stress Conditions

**Figure 3:**
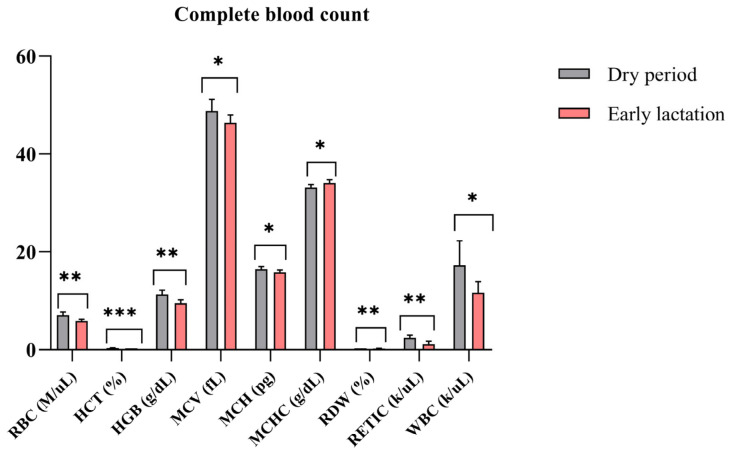
Differences in blood biochemical parameters, including the total number of erythrocytesRBC), hematocrit (HCT) value, i.e., erythrocyte ratio of total blood volume, hemoglobin concentration (HGB), mean erythrocyte volume in total sample (MCV), mean hemoglobin volume per red blood cell (RBC) count (MCH), mean hemoglobin concentration of erythrocytes (MCHC), the degree of variation in size of the erythrocyte population (RDW), reticulocyte count (RETIC), and the total number of leukocytes (WBC count), during heat stress (HS) conditions between the dry and early lactation periods of Holstein cows. Statistical significance is considered at a *p*-value less than 0.05 (*p* < 0.05). *** *p* < 0.0001; ** *p* < 0.001; * *p* < 0.01.

Significant differences were observed between the dry and early lactation periods for several complete blood count (CBC) parameters in Holstein dairy cows (Figure 3). The red blood cell (RBC) count was significantly lower during the early lactation period at 5.89 ± 0.13 M/µL than during the dry period at 7.08 ± 0.26 (*p* = 0.0014). Similarly, hematocrit (HCT) values were significantly reduced during the early lactation period (0.27 ± 0.01%) relative to the dry period (0.34 ± 0.01%, *p* = 0.0004). In addition, hemoglobin (Hb) levels followed this trend, with values of 9.56 ± 0.26 g/dL in the early lactation period versus 11.34 ± 0.33 g/dL in the dry period (*p* = 0.0011). Mean corpuscular volume (MCV) and mean corpuscular hemoglobin (MCH) showed a significant increase from the early lactation period (46.34 ± 0.61 fL; 15.83 ± 0.17 pg) to the dry period (48.76 ± 0.89 fL, *p* = 0.0451; 16.41 ± 0.20 pg, *p* = 0.0458). Moreover, mean corpuscular hemoglobin concentration (MCHC) decreased significantly from the early lactation period (34.09 ± 0.24 g/dL) to the dry period (33.17 ± 0.22 g/dL) (*p* = 0.0159). The degree of variation in red blood cell size, red cell distribution width (RDW), and reticulocyte count (RETIC) were both notably lower during the early lactation period, with RDW at 0.24 ± 0.01% and RETIC at 1.13 ± 0.23 k/µL, compared with the dry period at 0.26 ± 0.00% (*p* = 0.0018) and 2.43 ± 0.23 k/µL (*p* = 0.0018), respectively. In addition, the total leukocyte count (white blood cell, WBC) was significantly higher in the dry period (17.22 ± 1.91 k/µL) compared with the early lactation period (11.66 ± 0.88 k/µL).

### 3.3. Comparison of Heat Shock Protein (HSP) Levels Between Dry and Early Lactation Under Heat Stress Conditions

**Figure 4:**
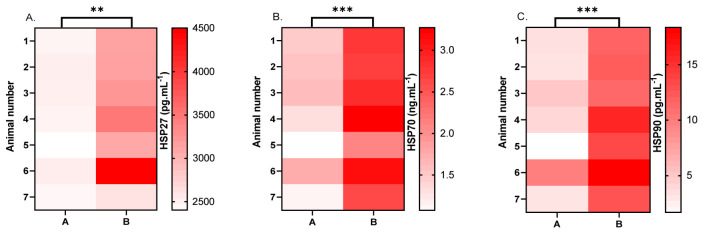
Blood serum concentrations of (**A**) heat shock protein (HSP)27, (**B**) HSP70, and (**C**) HSP90 under heat stress (HS) conditions in the dry and early lactation periods of dairy cows. Differences between the dry and early lactation periods were considered significant at *p* < 0.05. Significance levels are denoted as *** *p* < 0.0001 and ** *p* < 0.001.

Blood samples were collected once from each animal at each physiological stage, and concentrations of HSP27, HSP70, and HSP90 were compared between the dry period and early lactation under heat stress conditions. The measured values in the dry period were 2489.77 ± 18.92 pg mL^−1^, 1.44 ± 0.10 ng mL^−1^, and 4.52 ± 1.00 pg mL^−1^, respectively, whereas in early lactation under heat stress they were 3329.49 ± 218.42 pg mL^−1^, 2.79 ± 0.14 ng mL^−1^, and 13.71 ± 0.95 pg mL^−1^, respectively. Overall, the concentrations of HSP27, HSP70, and HSP90 were significantly higher during early lactation under heat stress compared with the dry period (*p* < 0.05; Figure 4A–C).

### 3.4. Comparison of Rumen Fermentation Parameters Between Dry and Early Lactation Under Heat Stress Conditions

**Figure 5:**
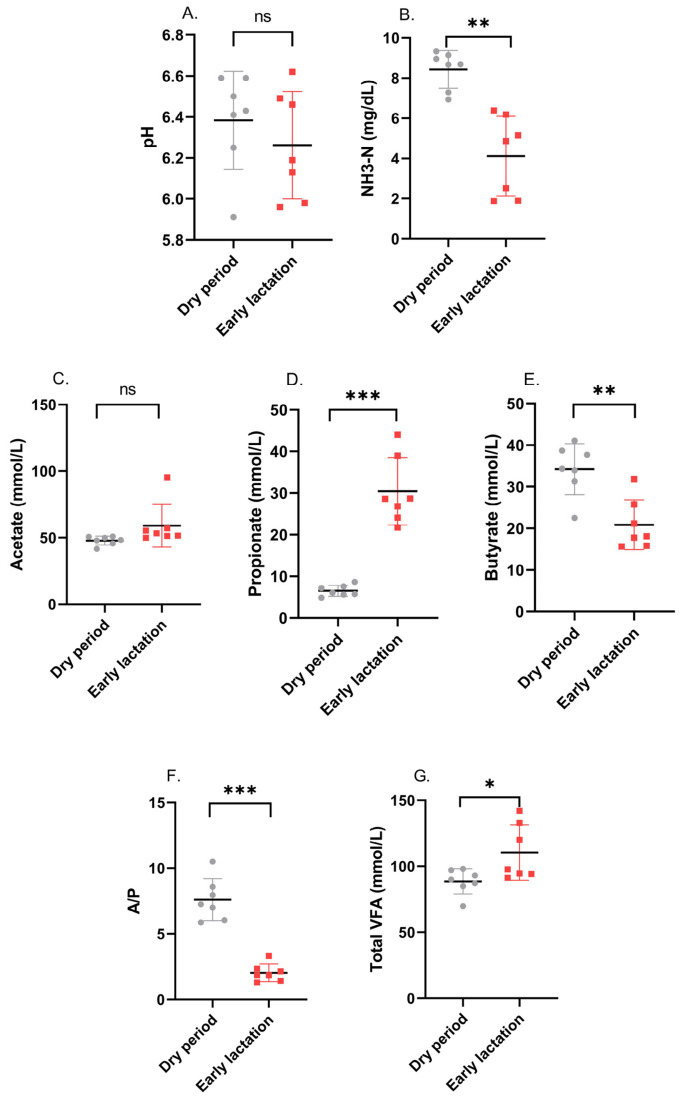
Statistically significant differences in rumen fermentation parameters during heat stress (HS) conditions between the dry and early lactation periods of dairy cows are shown, including (**A**) pH, (**B**) ammonia nitrogen (NH_3_-N), (**C**) acetate, (**D**) propionate, (**E**) butyrate, (**F**) acetate-to-propionate (A/P) ratio, and (**G**) total volatile fatty acids (VFA). Values are presented for clarity. VFA, volatile fatty acids. Differences between the dry and early lactation periods were considered significant at *p* < 0.05. Significance levels are indicated as *** *p* < 0.0001, ** *p* < 0.001, * *p* < 0.01, and ns (not significant) *p* > 0.05.

Under heat stress conditions, rumen fermentation parameters in Holstein cows during the dry and early lactation periods are shown in Figure 5A–G. Although the difference in pH values was not statistically significant (*p* > 0.05) between the two periods (dry period: 6.38 ± 0.09; and early lactation: 6.26 ± 0.10, the pH value was lower during the early lactation period than in the dry period. NH_3_-N concentrations were lower during the early lactation period at 4.12 ± 0.75 mg/dL than in the dry period at 8.43 ± 0.35 mg/dL. Additionally, acetate concentrations did not show a significant difference during those periods (*p* > 0.05) between the dry period (47.87 ± 1.25 mmol/L) and the early lactation period (59.10 ± 6.10 mmol/L). However, the concentrations of propionate, butyrate, and total VFA and the acetate-to-propionate (A/P) ratios were significantly different (*p* < 0.05) between the dry-period (6.51 ± 0.50 mmol/L, 34.22 ± 2.31 mmol/L, 88.60 ± 3.62 mmol/L, and 7.60 ± 0.61, respectively) and the early lactation period (30.42 ± 3.06 mmol/L, 20.87 ± 2.26 mmol/L, 110.39 ± 7.94 mmol/L, and 2.04 ± 0.26, respectively).

### 3.5. Comparison of Ruminal Bacterial Diversity Between Dry and Early Lactation Under Heat Stress Conditions

**Figure 6:**
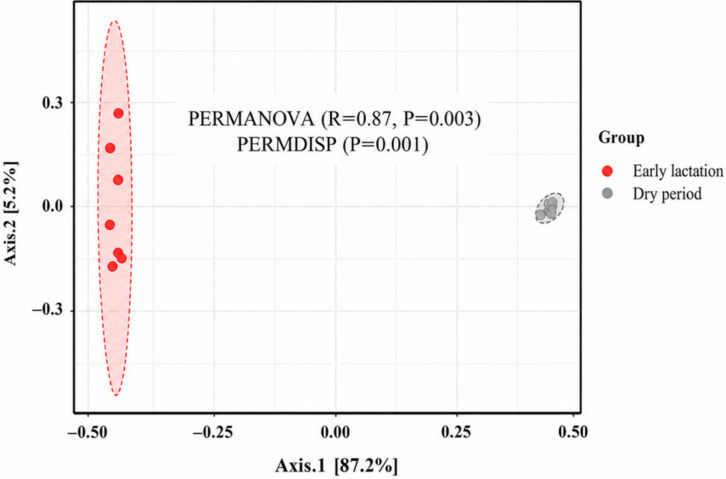
Beta diversity of the rumen bacterial community in dry and early lactation cows based on principal coordinate analysis (PCoA). Each point represents an individual sample. PERMANOVA (*R* = 0.87, *p* = 0.003) and PERMDISP (*p* = 0.001) indicate significant differences between the groups.

Principal coordinate analysis (PCoA) was performed based on the Bray–Curtis index to compare the bacterial communities between the dry and early lactation periods in dairy cows under HS conditions (Figure 6). The PCoA results revealed a clear separation of bacterial communities between samples from the two groups. The PERMANOVA results indicated a significant difference between the two groups (*p* = 0.003), and PERMDISP results confirmed a significant difference in dispersion between the groups (*p* = 0.001).

**Figure 7:**
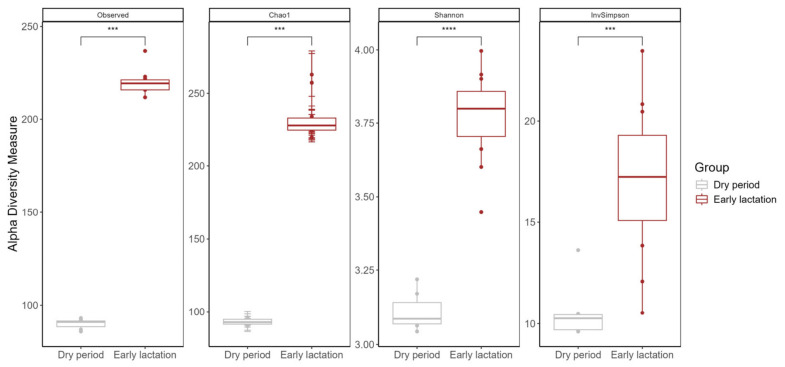
Boxplots depicting the distribution of the alpha-diversity metrics of ruminal samples during heat stress (HS) conditions in the dry and early lactation periods. Significance is assigned based on the results of the non-parametric Kruskal—Wallis test, followed by the pairwise Wilcoxon rank sum test. Cows belonged to either the dry or early lactation stages under heat stress conditions. Differences between the dry and early lactation periods were considered significant at *p* < 0.05. Significance levels are denoted as **** *p* < 0.00001 and *** *p* < 0.0001.

The results of the alpha diversity index analysis are shown in Figure 7. Significant differences were observed between the α-diversity, Chao1, Shannon, and Simpson indices between the dry and early lactation periods for the observed species, as determined by *t*-test and ANOVA.

**Figure 8:**
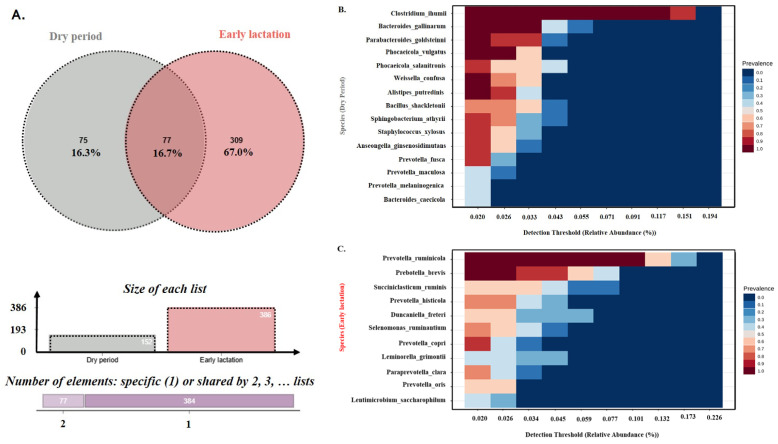
(**A**) Venn diagram of rumen bacteria under heat stress in the dry and early lactation periods. Both groups shared 77 (16.7%) species, whereas 75 (16.3%) and 309 (67.0%) species were unique to the dry and early lactation periods, respectively. (**B**) Detection threshold of core microbiome analysis relative abundance of 0.02% species of observed amplicon sequence variations ASVs during the dry period, and (**C**) detection threshold of core microbiome analysis at a relative abundance of 0.02% species of observed ASVs during the early lactation period. A blue-to-red color gradient is used to display bacterial community abundance across periods, with blue representing high abundance and red representing low abundance.

A total of 77 (16.7%) shared species were observed between the dry and early lactation periods under HS conditions, as illustrated in Figure 8A, whereas 75 (16.3%) and 309 (67.0%) unique species were observed during the dry and early lactation periods, respectively. The core microbiome analysis detection thresholds at a relative abundance of 0.02% species of observed ASVs during the dry and early lactation periods are shown in Figure 8B,C, providing a graphical representation of the differences in bacterial communities between the two periods under HS conditions.

### 3.6. Comparison of Rumen Bacterial Composition Between Dry and Early Lactation Under Heat Stress CONDITIONS

**Figure 9:**
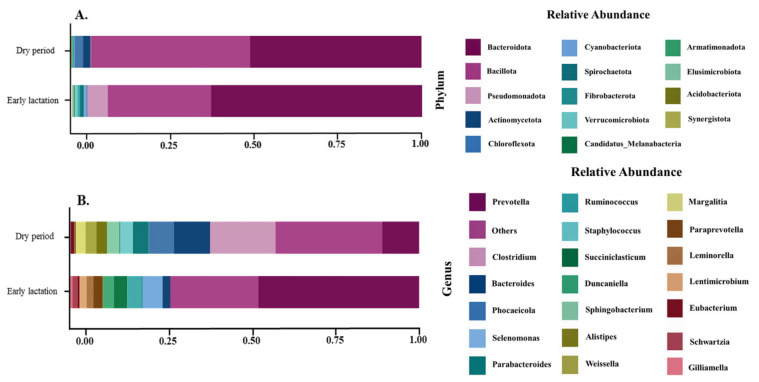
(**A**) Mean ruminal taxonomic profiles at the phylum level under heat stress during the dry and early lactation periods are shown. Stacked bar plots display the 15 most abundant phyla, each with an average relative abundance of at least 2%. (**B**) Mean ruminal taxonomic profiles at the genus level under heat stress during the dry and early lactation periods are also presented. The stacked bar plots illustrate the 20 most abundant taxa at an average relative abundance of at least 2%.

In this study, the effects of heat stress (HS) during the dry and early lactation periods on ruminal microbiota composition were evaluated in dairy cows. Ruminal bacterial communities were taxonomically classified at both the phylum and genus levels to determine relative abundance patterns. The taxonomic analysis identified three dominant phyla, each with an average relative abundance of at least 2%, in the rumen across the dry and early lactation periods, namely Bacteroidota, Bacillota, and Pseudomonadota. At the genus level, four predominant genera with an average relative abundance of ≥2% were observed during both periods: *Prevotella*, *Bacteroides*, *Ruminococcus*, and *Succiniclasticum* (Figure 9A,B).

**Figure 10:**
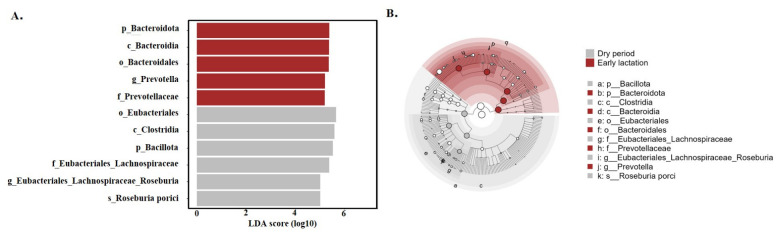
Linear discriminant analysis effect size (LEfSe) analysis was used to assess the effect of heat stress (HS) on ruminal bacterial communities during the dry and early lactation periods. (**A**) Differential taxa are shown with LDA scores (log10-transformed), where bar length indicates effect size and colors represent group-specific enrichment (LDA score > 2.0). (**B**) The cladogram displays differentially abundant taxa in a phylogenetic tree, with branch colors indicating the group with higher relative abundance.

LEfSe analysis was performed to identify the specific effects of heat stress (HS) on ruminal microbiota during the dry and early lactation periods, using an LDA score threshold of >2.0. During the early lactation period, five taxa were significantly enriched, including Bacteroidota, Bacteroidia, Bacteroidales, *Prevotella*, and Prevotellaceae. In contrast, during the dry period, six taxa were significantly enriched, including Eubacteriales, Clostridia, Bacillota, Eubacteriales_Lachnospiraceae, *Eubacteriales_Lachnospiraceae_Roseburia*, and *Roseburia porci* (Figure 10A,B).

**Figure 11:**
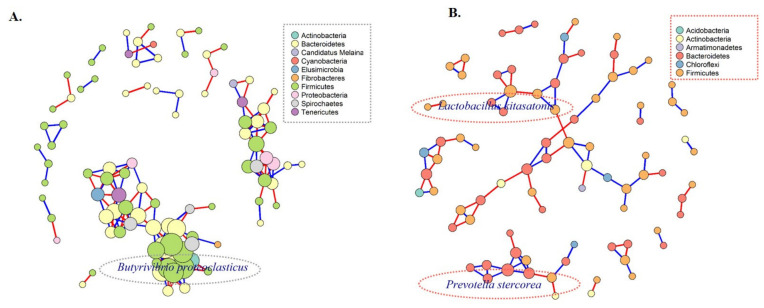
Microbial co-occurrence networks of ruminal microbiota under heat stress (HS) in dairy cows during (**A**) the dry period and (**B**) early lactation. Networks illustrate microbial interactions under HS conditions. Analyses were based on a prevalence threshold of 0.75, with strong correlations (|r| ≥ 0.8, *p* < 0.05). Nodes represent taxa and edges indicate correlations (blue, positive; red, negative).

Microbial co-occurrence networks were constructed to examine interactions among ruminal microbial species and to evaluate the potential impact of heat stress (HS) during the dry and early lactation periods on these relationships. A prevalence filtering threshold of 0.75 was applied to reduce sparsity and retain taxa consistently present across samples, and only strong correlations (absolute value ≥ 0.8) with statistical significance (*p* < 0.05) were included to focus on robust microbial associations. The network constructed from samples collected during the dry and early lactation stages showed a high level of complexity, comprising 97 species linked by 122 edges, with a similar distribution of positive and negative interactions. A relatively high diversity of microbial phyla was observed, with Bacillota and Bacteroidota being co-dominant. *Butyrivibrio proteoclasticus* emerged as a keystone species with 10 connections during the dry period, whereas 85 species were interconnected by 89 edges. During the early lactation period, *Lactobacillus kitasatonis* and *Prevotella stercorea* were the keystone species, each with six connections (Figure 11A,B).

## 4. Discussion

The study highlights substantial disparities in blood biochemical indicators between the dry period and early lactation phases in dairy cows under HS conditions. These metabolites may serve as early indicators for monitoring energy imbalance under heat stress in dairy production systems. Glucose levels decreased during early lactation, indicating increased energy demand associated with milk production. This reduction in glucose levels may indicate a heightened energy demands as milk production begins in cows [20]. In contrast, ketone concentrations were higher in early lactation compared with during the dry period. This elevation in the levels of ketone bodies suggests increased fat mobilization, a common metabolic adaptation to meet the energetic needs of lactating cows [21]. BUN levels demonstrated significant variations, being lower in early lactation than in the dry period. Ca levels were notably higher in the dry period, compared with during early lactation. Total protein concentrations significantly increased during early lactation, compared with in the dry period. This substantial rise in total protein levels indicates enhanced protein synthesis, reflecting the increased energy and nutritional requirements during lactation. The elevation in total protein concentration suggests that lactating cows undergo physiological adaptations to meet the substantial demands of milk production, which requires a continuous supply of amino acids [22]. During early lactation, AST levels were elevated in contrast to observed in the dry period. This notable increase in AST levels may indicate increased metabolic activity or potential tissue stress related to the physiological changes occurring during lactation. Elevated AST levels during early lactation are typically associated with liver function and muscle breakdown, reflecting the utilization of fat and protein reserves in cows to meet the increased energy requirements of milk production [23]. Bilirubin concentrations were higher during the early lactation period, compared with those during the dry period. This variation in bilirubin levels suggests a physiological shift during the transition from the dry period to lactation. Cholesterol levels displayed an inverse relationship, being notably lower during the early lactation period than in the dry period. Increased body temperatures and stress levels can impair liver function, which is crucial for cholesterol synthesis [24]. Conversely, phosphorus levels were higher in early lactation than those in the dry period. This increase may be linked to enhanced energy and protein metabolism, which are essential for supporting lactation. Under HS conditions, phosphorus metabolism can also be affected as it plays a vital role in energy production and cellular function. Increased metabolic activity during lactation requires relatively high phosphorus availability to support high milk production and fertility [25]. Mg levels remained relatively stable, both during early lactation and during the dry period. These findings demonstrate significant metabolic changes as dairy cows transition from the dry period to the early lactation period, characterized by alterations in energy and protein metabolism. These shifts highlight the importance of proper nutritional management to address increased physiological demands and ensure the overall health and productivity of lactating cows.

We observed notable differences in CBC parameters between the dry and early lactation periods in Holstein dairy cows, particularly under HS conditions. The results revealed a significant decrease in RBC count during the early lactation period, with compared with in the dry period. In addition, HCT levels showed a marked decline during the early lactation period compared with in the dry period. This substantial decrease in HCT levels suggests a reduction in blood volume concentration, which could impair oxygen transport capacity during a period of high metabolic demand because of milk production. The lower HCT values observed during the early lactation period than those in the dry period may be attributed to changes in the plasma volume and physiological responses to the increased energy and nutrient requirements of lactation. This decrease in HCT levels may reflect an expansion in blood plasma volume to support milk secretion, resulting in a dilutional effect on RBC mass [26]. In addition, the results revealed significant increases in MCV and MCH from early lactation to the dry period. These elevations in MCV and MCH may represent an adaptive mechanism where the size and hemoglobin content of RBCs are modified to enhance their oxygen-carrying capacity during periods of heightened metabolic demands associated with lactation. A significant reduction in MCHC, which represents the average hemoglobin concentration in RBCs, was observed from in the early lactation period to during the dry period. Hematological responses to HS varied considerably between these two periods. Cows in the early lactation stage typically exhibit more pronounced alterations in RBC counts, hemoglobin levels, and MCHC than those in cows in the dry stage, suggesting a more intense reaction to HS [20]. Studies have indicated that both RDW and RETIC are substantially lower during the early lactation period than during the dry period. RDW measured during the early lactation period in contrast to, demonstrating high statistical significance [27]. Likewise, RETIC showed a marked decrease in the early lactation period, with values of compared with in the dry period. These findings indicate a physiological adaptation in RBC dynamics during lactation, as evidenced by significant changes in RDW and reticulocyte levels [27]. The total WBC count in dairy cows showed a substantial increase from in the early lactation period to in the dry period, which suggests a notable physiological shift in the immune response of cows as they transition from lactation to the dry period [28].

The data indicated a persistent presence of HSPs across both stages, with notable fluctuations, particularly when comparing both stages under HS conditions. HSP expression profiles may be explored as potential biomarkers for heat stress adaptation in Holstein dairy cattle. The statistical analysis revealed that the disparities in protein levels between these two stages were statistically significant (*p* < 0.05), emphasizing the physiological shifts that occur as dairy cows transition from the dry period to the early lactation period. The dry period was marked by a considerable decrease in metabolic activity and physiological stress compared that during the early lactation period, resulting in lower HSP levels in the dry period than those in the early lactation period. During the dry period, cows are not subjected to the same physiological demands as during lactation, leading to a reduced need for protective proteins such as HSPs [29]. The elevated serum concentrations of these proteins suggest that dairy cows experience substantial physiological stress during early lactation, particularly when subjected to HS, which highlights the need for effective management strategies to alleviate the negative effects of HS on cow health and milk production. The increase in HSP27, HSP70, and HSP90 levels during early lactation represents an adaptive response to the challenges presented by both lactation and environmental stressors, highlighting the importance of monitoring the welfare of cows during this period and implementing effective management practices in dairy herds [30]. The substantial differences in HSP levels between the dry and early lactation periods can be attributed to decreased physiological stress during the dry period, heightened metabolic requirements during the early lactation period, and hormonal changes that enhance HSP expression in response to environmental and physiological challenges.

Studies on rumen fermentation characteristics in Holstein cows experiencing HS during their dry and early-lactation periods offer crucial insight into their metabolic adjustments. Although pH levels showed no significant variation between these phases a slight downward trend was observed during the early lactation period. This minor decrease is consistent with the results of previous studies, indicating that the transition to lactation often involves dietary changes and shifts in rumen microbiota, potentially affecting ruminal pH [31]. The marked decrease in NH_3_-N concentrations during the early lactation period compared with that in the dry period indicates enhanced amino acid utilization and protein synthesis, aligning with research showing that early-lactating cows typically experience increased microbial protein production because of higher energy intake and improved rumen fermentation efficiency during this period. Moreover, reduced NH_3_-N levels can help mitigate the risk of subacute ruminal acidosis (SARA), which is essential for maintaining rumen health during early lactation [32]. In the present study, acetate concentrations in dairy cows remained relatively constant during the transition from the dry period to the early lactation period, measuring in the dry period and rising to in the early lactation period. This stability in acetate levels is noteworthy, as acetate is primarily used for milk fat synthesis. However, this apparent constancy may mask the simultaneously occurring underlying metabolic changes during the transition [33]. Conversely, propionate levels showed a significant surge, from during the dry period to in the early lactation period. This increase in propionate levels is crucial, as propionate functions as a primary glucose precursor, vital for addressing the increased energy requirements linked to milk production during the early lactation [34]. The higher butyrate concentration observed during the dry period may be associated with a relatively higher forage proportion in the diet, which promotes the activity of fiber-degrading bacteria such as *Butyrivibrio*. In contrast, the shift toward a higher concentrate diet during early lactation favors propionate production, resulting in a reduction in butyrate levels. Moreover, the A/P ratio underwent a considerable reduction, from in the dry period to during the early lactation period. A reduced A/P ratio is commonly associated with enhanced propionate production, which boosts the gluconeogenic potential. This result is particularly relevant, as the metabolic adaptations of ruminants during the early lactation phase enable them to efficiently use propionate for glucose synthesis, a necessary adjustment, considering the energy deficits often encountered in the early lactation phase because of insufficient feed intake [35]. The observed decline in the A/P ratio is linked to dietary shifts favoring high-concentrate feeds, which boost propionate production relative to acetate [36]. Furthermore, butyrate concentrations notably decreased from in the dry period to the early lactation period. Although butyrate is important for rumen epithelial health and energy mobilization, a reduction in its concentrations may indicate changes in fermentation patterns due to dietary alterations during lactation. Butyrate concentrations notably decreased from the dry period to the early lactation period. Butyrate concentrations can vary widely depending on dietary forage-to-concentrate ratio and rumen fermentation patterns, and higher values may be associated with greater fiber fermentation activity during high-forage diets in the dry period [37]. The overall rise in total VFA concentrations, from in the dry period to in the early lactation period, indicates increased fermentation activity, crucial for maximizing nutrient absorption and energy availability for milk production [38]. The variations in rumen fermentation parameters between the dry and early lactation phases highlight the complex metabolic adjustments in Holstein cows under HS.

These microbial shifts may be targeted in future nutritional strategies to improve rumen efficiency and heat stress resilience. PCoA based on the Bray–Curtis index, was performed to compare the bacterial communities of dry-period and early-lactating cows during HS. This helps visualize differences in community composition [39]. The analysis revealed a clear separation between samples from the two groups, indicating distinct microbial profiles associated with different physiological states. PERMANOVA results indicated a significant difference between the groups (*p* = 0.003), suggesting that variations in community structure are not because of random chance but are impacted by the differences in lactation and HS conditions. Furthermore, PERMDISP results confirmed a significant difference in dispersion between the two groups (*p* = 0.001), indicating that the variability within each group also differed significantly [40]. These results collectively highlight the impact of both physiological state and environmental stress on ruminal bacterial community composition in dairy cows, which has crucial implications for their health and productivity under challenging conditions. The combined use of PCoA, PERMANOVA, and PERMDISP provides a robust framework for analyzing and interpreting the complex interactions in microbial communities impacted by environmental factors in dairy cows.

Alpha diversity indices are critical metrics for quantifying the species diversity within a given sample or community. In the context of microbiomes, these indices provide valuable insights into the health and stability of the microbiota. The alpha diversity can be measured using various indices, including the Shannon, Simpson, and Chao1 indices. Each of these indices considers different aspects of diversity providing a comprehensive view of community structure and health [41]. In this present study, significant differences in alpha diversity indices were observed between the dry and the early lactation periods of dairy cows. The observed species richness, calculated as the total number of unique species identified, served as a fundamental measure of diversity. Higher microbial richness values during the early lactation period than those in the dry period suggest increased microbial diversity, which may be beneficial for the metabolic health of dairy cows [42], In addition, the Chao1 index, an estimator that accounts for unseen species in the observed data, demonstrated significant variability between the two periods. This metric is crucial for elucidating the potential for observing addition species in the studied community, thereby reflecting the ecological balance and resilience of the microbial community during early lactation [42].

HS significantly affects the microbial composition in dairy cows, particularly during critical production periods such as the dry and early lactation periods. In this study, we identified 77 shared species (16.7%) between these two periods, emphasizing the stability of certain microbial communities despite varying physiological demands. Additionally, 75 unique species (16.3%) were observed exclusively during the dry period, while 309 unique species (67.0%) were observed during the early lactation period. This disparity highlights the dynamic nature of the ruminal microbiome, which adapts to metabolic changes associated with lactation demands, feeding practices, and environmental stressors [43]. A core microbiome was used to explore differences in ruminal bacterial communities observed during HS across these two critical periods. This analytical approach focused on the top 15 bacterial communities in the dry period, and the top 11 in the early lactation period, providing comprehensive insights into shifts in microbial populations under thermal stress. Such shifts in microbial populations can have considerable implications for the health and productivity of dairy cows, as microbial communities play a crucial role in nutrient digestion and metabolism [9]. These results are visually represented in Figure 7 which provide a graphical depiction of the variations in bacterial communities influenced by HS during these critical physiological phases [11].

In this study, we investigated whether HS during the dry and early lactation periods affected the composition of ruminal microbiota. HS is known to significantly impact the microbial community structure in ruminants, altering feed intake and metabolic processes, which ultimately affect microbiota composition throughout critical production periods [11]. We taxonomically classified ruminal bacteria at both the phylum and genus levels to assess their relative abundances within the microbiota composition. The taxonomic classification of ruminal bacteria, particularly using 16S rRNA sequencing, enables a detailed elucidation of microbial diversity and their relative abundances, which is essential for interpreting how environmental stresses influence ruminal function [44]. The taxonomic analysis revealed three predominant phyla, each with an average relative abundance of at least 2%, in the rumen during the dry and early lactation periods: Bacteroidota, Bacillota, and Pseudomonadota. In particular, Bacteroidota and Bacillota are recognized as the primary bacterial phyla involved in the breakdown of complex carbohydrates within the rumen, which is vital for ruminants to efficiently utilize fibrous feeds. The members of Bacteroidota, such as *Prevotella*, have been shown to effectively ferment polysaccharides, contributing to VFA production and energy supply for the animal [45]. Bacteroidota thrives on high-fiber diets, indicating their importance in ruminant nutrition, while Bacillota also contains many important fiber-degrading species [46]. Fermentation mediated by both Bacteroidota and Bacillota generates VFAs that provide energy to dairy cows and support their overall metabolic health [47]. The pathophysiology of SARA involves a significant drop in ruminal pH because of excessive acid production, often caused by an overabundance of rapidly fermentable carbohydrates that skew the microbiome balance towards a higher ratio of Pseudomonadota relative to Bacteroidota and Bacillota [48]. At the genus level, four main genera, each with an average relative abundance of at least 2%, were identified during the two stages: *Prevotella*, *Bacteroides*, *Ruminococcus*, and *Succiniclasticum*. The genera *Prevotella* and *Ruminococcus* are particularly important as they are involved in the breakdown of complex carbohydrates and facilitate energy extraction from plant materials. Their abundance is often correlated with dietary composition and overall ruminal health, with *Prevotella* being prominent in environments rich in soluble carbohydrates. The results of this insightful analysis highlight the significance of monitoring microbial communities during the critical peripartum period in dairy cows, particularly under the stresses posed by heat [11].

LEfSe was used to elucidate the specific effects of HS on ruminal microbiota during the dry and early lactation stages. In the early lactation phase, five taxa, Bacteroidota, Bacteroidia, Bacteroidales, *Prevotella*, and Prevotellaceae were significantly enriched. This finding suggests their crucial role in digestion and nutrient absorption processes of lactating dairy cows, potentially enhancing their metabolic efficiency during this critical phase [11]. Conversely, during the dry period, six distinct taxa enriched: Eubacteriales, Clostridia, Bacillota, Eubacteriales_Lachnospiraceae, *Eubacteriales_Lachnospiraceae_Roseburia*, and *Roseburia porci*. This highlights the remarkable adaptability of the microbiota, showing how these microbial populations respond variably to environmental stressors such as heat, during different physiological phases. Such adaptability suggests a dynamic interplay between the host and its microbiota, enabling for adjustments in rumen ecology, which is crucial for maintaining metabolic homeostasis [49]. The specific enrichment observed in the microbial taxa, particularly those under the orders Eubacteriales and Clostridia, indicates their crucial involvement in fermentation and metabolic processes occurring within the rumen. These taxa produce important fermentation products, such as VFAs, vital for energy metabolism in ruminants. The increase in these specific microbial populations suggests their significant role in enhancing the fermentation efficiency during the periods of thermal stress [50].

The investigation of microbial interactions through co-occurrence networks has provided insights into the complex relationships between ruminal species during the critical periods of dairy cow management, particularly in the context of HS during the dry and early lactation periods. To assess these interactions, a prevalence filtering threshold of 0.75 was applied, focusing on strong correlations (absolute value ≥ 0.8) that were statistically significant (*p* < 0.05). The resulting network displayed intricate connections, comprising 97 species interconnected by 122 edges. This complexity reflects the diverse microbial ecosystems present in the rumen, where both positive and negative interactions shape microbial dynamics. The observed co-dominance of Bacillota and Baecteroidota is consistent with the results of previous studies emphasizing their pivotal roles in fiber fermentation and overall ruminal function in dairy cows. During the dry period, *B. proteoclasticus* emerged as a keystone species, connecting to 10 different species. As the early lactation period commenced, *L. kitasatonis* and *P. stercorea* emerged as the key players, each linked to six other species. The impact of HS on these microbial associations is significant, as HS can alter the diversity and function of the rumen microbiota. HS disrupts the balance between various microbial populations, potentially leading to negative effects on overall rumen function and animal health [51]. The results of the microbial co-occurrence network analysis, emphasize the critical interplay between ruminal species and the cascading effects of HS during the dry and early lactation periods. The identification of key species, such as *B. proteoclasticus*, *L. kitasatonis*, and *P. stercorea,* highlights the need for maintaining a balanced microbiome for optimal ruminal function, essential for the health and productivity of dairy cows [9]. Differences in total mixed ration (TMR) composition between the dry and early lactation periods, particularly in neutral detergent fiber (NDF) and acid detergent fiber (ADF) levels, may have influenced rumen fermentation characteristics and microbial community profiles; therefore, the observed differences should be interpreted as a combined effect of dietary composition, physiological stage, and heat stress conditions. A formal power analysis was not conducted prior to the study; however, the sample size was determined based on animal availability under farm conditions and in accordance with similar published studies on dairy cows under heat stress. Milk yield data were not collected in this study; therefore, the relationship between the observed physiological, rumen fermentation, and microbial changes and actual milk production performance under heat stress could not be evaluated. A thermoneutral control group was not included in this study; therefore, it is not possible to fully distinguish whether the observed differences in blood biochemistry, hematological parameters, rumen fermentation, and microbial composition are solely attributable to heat stress or are also influenced by inherent physiological differences between dry and early lactation stages. However, limitations of this study include a small sample size and focus on data from a single season. Further research across multiple seasons and in relatively large herds could enhance our understanding. Overall, these findings demonstrate that heat stress during the transition period is associated with substantial metabolic and microbial adjustments in dairy cows. Understanding these stage-specific responses provides valuable information for improving nutritional and management strategies aimed at reducing the negative effects of heat stress and supporting rumen function, health, and productivity in dairy cattle. Future studies with larger animal populations and multi-season observations will help clarify the long-term implications of heat stress on rumen microbial ecology and dairy cow performance, and support more effective management strategies for dairy production under HS conditions.

## 5. Conclusions

Heat stress induced distinct metabolic, hematological, ruminal fermentation, and microbial alterations in Holstein dairy cows between the dry and early lactation periods. Early lactation cows showed lower glucose and cholesterol levels, alongside higher ketone and AST concentrations, indicating increased metabolic demand. In addition, rumen fermentation and microbial composition shifted toward enhanced propionate production and enrichment of carbohydrate-utilizing taxa such as *Prevotella*. Overall, these findings demonstrate stage-specific physiological and microbial adaptations to heat stress in dairy cows during the summer period in Korea.

## Figures and Tables

**Table 1:** Ingredients and chemical composition (%) of total mixed ration (TMR) fed to lactating Holstein cows during their dry and early lactation periods (DM basis).

Item	Composition (%)	
	Dry	Early Lactation
Ingredients (%)		
Lupine seed	7.50	7.50
Whole cottonseed	10.73	10.79
Tall fescue	28.67	29.35
Corn hull	16.09	16.10
Corn flake	10.40	10.82
Corn silage	11.70	10.76
LWheat bran	14.02	14.01
Salt	0.33	0.33
Vitamin-mineral mix ^1^	0.33	0.33
Limestone	0.17	0.17
Calcium phosphate	0.17	0.17
Chemical composition (% as DM basis)		
Crude protein	14.12	13.05
Crude fat	2.71	1.83
Crude fiber	23.98	24.47
Crude ash	10.24	6.86
Calcium	1.01	0.94
Phosphorus	0.29	0.33
Acid detergent fiber (ADF)	23.45	15.27
Neutral detergent fiber (NDF)	52.68	32.44

^1^ Vitamin-mineral mix contained vit. A 2,650,000 IU, vit. D3 530,000 IU, vit. E 1050 IU; niacin 100 10,000 mg; Mn 4400 mg; Zn 4400 mg; Fe 13,200 mg; Cu 2200 mg; iodine 440 mg; and Co 440,101 mg/kg of Grobic-DC were provided by Bayer Health Care.

## Data Availability

The datasets generated and/or analyzed during the current study are available from the corresponding author upon reasonable request.

## Abbreviations

The following abbreviations are used in this manuscript:

ADF	Acid detergent fiber
A:P	Acetate:propionate
AST	Aspartate aminotransferase
ASV	Amplicon sequence variants
BUN	Blood urea nitrogen
Ca	Calcium
DM	Dry matter
DMI	Dry matter intake
DNA	Deoxyribonucleic acid
HCT	Hematocrit value: erythrocyte ratio of total blood value
HS	Heat stress
HSP	Heat shock protein
MCH	Mean hemoglobin volume per RBC count
MCHC	Mean hemoglobin concentration of erythrocytes
MCV	Mean erythrocyte volume in total sample
Mg	Magnesium
MUN	Milk urea nitrogen
NDF	Neutral detergent fiber
NH_3_-N	Ammonia nitrogen
LDA	Linear discriminant analysis
LEfSe	Linear discriminant analysis effect size
PCoA	Principal coordinates analysis
PERMANOVA	Permutational multivariate analysis of variance
PERMSISP	Permutational analysis of multivariate dispersions
RBC	Total number of erythrocytes
RDW	Red cell distribution width
RETIC	Reticulocyte count
rRNA	Ribonucleic ribonucleic acid
SARA	Subacute ruminal acidosis
SAS	Statistical analysis systems
SNF	Solid non-fat
THI	Temperature humidity index
TMR	Total mixed ratio
VFA	Volatile fatty acid
WBC	Total number of leukocytes
